# Flavor and Aroma Analysis as a Tool for Quality Control of Foods

**DOI:** 10.3390/foods10020224

**Published:** 2021-01-22

**Authors:** Ángel Calín-Sánchez, Ángel A. Carbonell-Barrachina

**Affiliations:** Research Group “Food Quality and Safety”, Department of Agro-Food Technology, Escuela Politécnica Superior de Orihuela (EPSO), Universidad Miguel Hernández de Elche (UMH), 03312 Orihuela, Alicante, Spain; angel.carbonell@umh.es

The aroma composition of foods has been the subject of considerable research in recent years. It is well known that the presence of volatile compounds and their composition determine the specific aroma of foods and the flavor of the resulting products. The composition and concentrations of volatile compounds depend on many factors including climatic and soil conditions, seasonal variation, agronomical practices and processing. On the other hand, sensory analysis is used to quantitatively determine the intensities of the main sensory properties and attributes of food as well as determine the preference. Such analysis requires the use of a trained panel and regular consumers.

Food quality constitutes the main concern of regular consumers with regard to sensory quality and economic factors. Farmers and manufacturers have made great efforts to achieve consumers’ considerations; however, the requirements in terms of production optimization due to environmental deficiencies and energy saving still need the role of science and innovation. Accordingly, and within the idea and objective of bringing together original studies dealing with the aroma profile and sensory quality of foods, we edit this Special Issue on “Flavor and Aroma Analysis as a Tool for the Quality Control of Foods”. From the twelve published papers, four main research topics were covered: (i) the effect of agricultural practices, (ii) the effect of different processing and conditions, (iii) the characterization of foods and (iv) consumer perception and acceptability.

In the first topic dealing with the effect of different agricultural practices on the quality of fruits, two papers can be categorized [1,2]. The first study by Noguera-Artiaga et al. [1] compares the different irrigation regimes (deficit, moderate and severe) of different pistachio cultivars and rootstocks. This study was conducted in Spain, a Mediterranean country with long periods of water scarcity. The results demonstrated that the application of a moderate deficit of irrigation during pistachio cultivation led to pistachios with the same morphological properties, total polyphenol content, antioxidant activity, volatile composition and sensory properties as pistachios obtained using full irrigation. Moreover, moderately irrigated pistachios led to the obtaining of a better profile of fatty acids and were the sample preferred by international consumers. On the contrary, when the deficit irrigation was severe, pistachio nuts had the lowest antioxidant activity, the lowest total polyphenols content and were the least preferred samples by consumers. In the case of pistachios obtained using different rootstocks, *P. integerrima* led to pistachio nuts with the highest weight, the lowest content of sucrose and better functional properties than *P. atlantica* and *P. terebinthus*. These results demonstrated that it is possible to save irrigation water in pistachio farming with a low environmental and economic cost and leading to pistachio nuts with same or even better quality attributes. The second study is by Aguilar-Hernández et al. [2], which was performed in order to determine whether the volatile profile of lemon peel oil was affected by the rootstock. The selected varieties of *Citrus limon* used in this study were “Bétera”, “Verna”, “Fino 49”, “Fino 95” and “Eureka” grafted on to the rootstocks Forner-Alcaide Nº5 , Forner-Alcaide Nº13, Forner-Alcaide Nº517, *C. macrophylla* West and *C. aurantium* L. All of them reside in the European Union (BOE/04/12/2007) and were obtained by targeted hybridizations by Forner in IVIA (Valencia) [3]. The results demonstrated that the Forner-Alcaide rootstocks were the best rootstocks leading to a high content of volatile compounds followed by *C. aurantium* and *C. macrophylla*. The order of the total volatile contents was (in decreasing order): “Eureka” > “Bétera” > “Fino 95” > “Verna” > “Fino 49”. These results confirmed that a strong relationship exists between the rootstock/scion combinations and the concentration of volatile compounds in the lemon peel oil. Aroma volatiles should be considered key parameters for the determination of rootstock-induced effects.

In the second topic of studies of different processing and conditions, six manuscripts have been published [4,5,6,7,8,9]. The authors provide an overview of recent advances made in the field of food processing establishing the best processing conditions in order to obtain food based products with a high quality in terms of aroma, flavor and, consequently, consumer satisfaction. The first study of this second topic by Zhang et al. [4] describes the main volatile compounds and marker substances during the concentration of coconut jam assessed by headspace solid phase microextraction (HS-SPME) combined with gas chromatography-mass spectrometry (GC-MS). The results showed that the concentration process of coconut jam occurs in three stages. In the early stage, alcohols and esters are responsible for the aroma, while ketones are the main compounds at the middle stage. Finally, in the final sterilization stage, a variety of aroma compounds are produced and form the unique flavor of coconut jam. The stepwise increase of the furfural content is consistent with the inflection point of the change in aroma during the whole process of coconut jam concentration. The logistic model has a higher degree of fit, which can be used as a marker of aromas to monitor the concentration of the product. However, the mechanisms of action of certain aroma compounds released from the coconut jam are still unclear. The second study by Cano-Lamadrid et al. [5] developed a confectionary product exclusively based on natural ingredients. This manuscript describes the quality parameters and consumer acceptance of jelly candies based on pomegranate juice. This study is a consequence of an upward trend towards reducing or suppressing food additives as well as reducing the use of E-numbers in labels, thus providing clean label foods. To reach this aim, the authors report that the formulation consisting of 20% gelatin with pure “Mollar de Elche” pomegranate juice, 1% citric acid and a sugar addition led to the best results in terms of color, texture, antioxidant capacity and sensory attributes. The most valued quality parameter of pomegranate products, the red color (a* coordinate), was not negatively affected by the process of preparing the jellies. The same authors claim that still this novel confectionary product must be improved and propose further investigations related to (i) hydrating gelatin in pomegranate juice instead of water and (ii) optimizing the drying step. The third study by Gasinski et al. [6] performed an assessment of volatile compounds among other determinators of beers enriched with dotted hawthorn (*Crataegus punctate*) by two ways of addition, fruit or juice. The study indicated that the addition of hawthorn led to a beer with an increased total content of polyphenolic compounds and antioxidant activity. Beer with a fruit addition was characterized by the greatest number of volatile compounds, eight times higher than in the control sample. The enrichment of the beer by hawthorn fruit mostly increased the concentration of the volatile compounds characterized by fruity and sweet aromas. The results of a sensory analysis indicated that the addition of hawthorn fruit resulted in an improvement of such characteristics as taste, aroma, clarity and overall impression to a higher degree than the addition of hawthorn juice. Hawthorn fruit and its juice can be used as a complementary raw material in the production of beer to increase its biological activity and improve its taste and aroma. It can also contribute to a greater consumer interest in the product. The fourth study by Issa-Issa et al. [7] had as main goal the investigation of the volatile composition by HS-SPME/GC-MS and a sensory profile by a descriptive sensory analysis of novel smoothies prepared by blending fig, jujube or quince purée with pomegranate juices (cv. “Mollar de Elche” or “Wonderful”) at two ratios of purée to juice, 40:60 or 60:40. Twenty-three volatile compounds were present with the five predominant ones as follows: (i) 5-HMF; (ii) 3-hexen-1-ol; (iii) hexanal; (iv) 1-hexanol and (v) 3-octanone. Fig smoothies were reported to be sweet and had a flavor and volatiles related to fig, pomegranate and grape. Meanwhile, jujube products were bitter and had jujube and pear notes. Finally, quince smoothies were sour and had quince, apple and floral notes. Thus, the type of fruit used clearly determined the flavor of the final product. The smoothies prepared with “Mollar de Elche” pomegranate juice were characterized by having a high intensity of pear odor/aroma and consistency while “Wonderful” smoothies were characterized by a lower consistency and a more intense pomegranate aroma and sour flavor. However, further research is still needed to fully optimize these novel products. Two ways of improvement can be researched: (i) increasing pomegranate notes and (ii) avoiding undesirable compounds after the Maillard Reaction. The fifth study by Kwasnica et al. [8] describes the volatile composition and the sensory properties as quality attributes of fresh and dried hemp flowers (*Cannabis sativa* L.). Flowers of hemp are widely used in cosmetics, food and in the pharmaceutical industry. The drying process plays a key role in the retention of the aroma and also in the quality of the products. Seven variants of hemp flower drying including convection drying, vacuum-microwave drying and combined drying consisting of convective pre-drying followed by vacuum-microwave finishing drying were checked in this study. During the drying process, losses found in 93 analyzed volatiles ranged from 48% for combined drying to 15% for vacuum-microwave drying at 240 W, which was finally chosen as optimal for the retention of aroma-active compounds. In that variant, a significant decrease of β-myrcene was observed. From a sensory point of view, the best drying treatment was vacuum-microwave drying at 240 W because it produced dried samples most resembling the fresh material with high intensities of key sensory descriptors such as hemp flower ID, fresh vegetables, citrus, balsamic and anise and therefore this drying treatment and conditions can be recommended as the best option for hemp flower drying. The sixth and last manuscript of this second topic by Calín-Sánchez et al. [9] is a review manuscript that compared traditional and novel drying techniques and describes their effect on the quality of fruits, vegetables and aromatic herbs. The quality of dehydrated fruits, vegetables and aromatic herbs is a key problem closely related to the development and optimization of novel drying techniques. This review reported the weaknesses of common drying methods applied for fruits, vegetables and aromatic herbs and the possible options to improve the quality of dried products using different drying techniques or their combination. In general, drying led to a reduction in all studied parameters. However, the behavior of each plant material was different. On the whole, the optimal drying technique was different for each of the materials studied and specific conditions were recommended after a proper evaluation of the drying protocols. However, a novel or combined technique must assure a high quality of dried products. Furthermore, the term ‘quality’ must englobe energy efficiency and the environmental impact leading to the production of sustainably dried products.

In the third topic that groups manuscripts dealing with the characterization of foods based on instrumental (GC-MS) and sensory analysis, three original papers have been published [10,11,12]. The first study of this group by Valli et al. [10] describes a screening support for panel tests in order to classify olive oils according to their volatile profile. A sensory evaluation, carried out by panel tests, is essential for a quality classification of virgin olive oils but is time consuming and costly when many samples need to be assessed; sensory evaluation could be assisted by the application of screening methods. Rapid instrumental methods based on the analysis of volatile molecules might be considered interesting to assist the panel test through fast pre-classification of samples with a known level of probability, thus increasing the efficiency of quality control. With this objective, a headspace gas chromatography-ion mobility spectrometer (HS-GC-IMS) was used to analyze 198 commercial extra virgin, virgin and lampante olive oils by a semi-targeted approach. The promising models were useful to predict the quality grade and presence of three sensory defects (musty, rancid, fusty/muddy sediment) providing percentages of correctly classified samples in external validation from 67% to 95% for the quality grade prediction model and from 48% to 80%, for the presence of each of the aforementioned defects. However, additional investigations are needed before it can be implemented commercially; furthermore, to test the performance of this approach, inter-laboratory tests involving independent laboratories will be carried out in the future. The second study of this topic by Vichi et al. [11] led to the distinguishing of homozygous and heterozygous bitter genotypes in sweet almonds. Bitterness in almonds kernels is due to the presence of the cyanogenic glucoside amygdalin, which undergoes enzymatic hydrolysis by β-glucosidases upon the disruption of tissues to form glucose, hydrogen cyanide and benzaldehyde [13]. The results demonstrated the association between sweet almonds’ genotype and a few volatile metabolites and provided for the first time chemical markers to discriminate between homo- and heterozygous sweet almonds. In particular, the amount of benzaldehyde, assessed by a simple, rapid, automatable and affordable technique such as SPME-GC-MS, allowed the differentiation between the homo- and heterozygous samples analyzed in the study and to tentatively classify almond kernels with an unknown genotype. The third and last research of this topic by Romero-Medina et al. [12] characterized a traditional Mexican corn beer by both sensory and instrumental analysis. The main objective of this study was to understand how the use of pigmented corn malt influences the chemical composition and sensory characteristics of beers. The authors demonstrated for the first time that among the groups of volatile compounds, ketones, terpenes and phenol volatiles as well as the presence of anthocyanins appeared as relevant criteria for the differentiation of corn beers. Moreover, the study of the relationship between the sensory attributes and the chemical parameters elucidated the effect of each type of malt (red corn, blue corn and barley malt) on the chemical parameters and their association with the sensory attributes. Finally, they declared that the sensory characteristics of these beers may carry the acceptance or rejection of consumers needs to be further investigated.

The last topic is composed by only one manuscript and is related to the sensory perception of novel fruit based products such as smoothies by a particular group of Spanish millennials. The work by Cano-Lamadrid et al. [14] applied Napping^®^, a descriptive sensory analysis and consumer studies in order to provide a new insight into the perception of smoothie products and comprehensive knowledge for the food industry to guide the design of new foods including functional and healthy fruit based products. The results showed that the descriptive sensory analysis and consumer studies preceded by the Napping^®^ test seemed to be an appropriate combination to optimize the formulation of novel fruit and vegetable smoothies. The key attributes controlling the overall liking were the adequate intensity of a sour taste and notes of mango, banana and peach. Nevertheless, it should strive to improve recipes of smoothies to increase the consumption of fruits and vegetables in this form, which is considered a simple supplement in a balanced diet. The results of the penalty analysis gave a good direction to optimize these types of smoothies by avoiding vegetable ingredients with earthy or strong vegetal notes. The research provided a series of practical tips for the food industry to understand consumer preferences, select raw materials and improve marketing strategies.

In summary, the twelve papers published in this Special Issue highlight a great part of the research activities in the field of food quality as a tool for quality control, aiming to characterize and improve the sensory, chemical, nutritional, health and physical quality of fruits and fruit based products. This Special Issue, with the worldwide trend toward foods for nutrition and health, further states the importance of multidimensional and multidisciplinary approaches as exemplified in the papers described above. Finally, most authors who have contributed to this issue state that further research in their topic is required in every one of the presented papers and this assures an exciting time for future studies.
